# Utilizing MALDI-TOF MS and LC-MS/MS to access serum peptidome-based biomarkers in canine oral tumors

**DOI:** 10.1038/s41598-022-26132-y

**Published:** 2022-12-14

**Authors:** Sekkarin Ploypetch, Janthima Jaresitthikunchai, Narumon Phaonakrop, Walasinee Sakcamduang, Sukanya Manee-in, Prapat Suriyaphol, Sittiruk Roytrakul, Gunnaporn Suriyaphol

**Affiliations:** 1 Department of Clinical Sciences and Public Health, Faculty of Veterinary Science, Mahidol University, 999 Phutthamonthon Sai 4 Road, Salaya, Phutthamonthon, Nakhon Pathom, 73170 Thailand; 2 Functional Ingredients and Food Innovation Research Group, National Center for Genetic Engineering and Biotechnology, National Science and Technology Development Agency, Pathum Thani, 12120 Thailand; 3 Division of Bioinformatics and Data Management for Research, Research Group and Research Network Division, Research Department, Faculty of Medicine Siriraj Hospital, Mahidol University, Bangkok, Thailand; 4 Biochemistry Unit, Department of Physiology, Faculty of Veterinary Science, Chulalongkorn University, Henri Dunant Road, Pathumwan, Bangkok, 10330 Thailand; 5 Center of Excellence for Companion Animal Cancer, Faculty of Veterinary Science, Chulalongkorn University, Bangkok, 10330 Thailand

**Keywords:** Cancer, Molecular biology

## Abstract

Tumors frequently found in dogs include canine oral tumors, either cancerous or noncancerous. The bloodstream is an important route for tumor metastasis, particularly for late-stage oral melanoma (LOM) and late-stage oral squamous cell carcinoma (LOSCC). The present study aimed to investigate serum peptidome-based biomarkers of dogs with early-stage oral melanoma, LOM, LOSCC, benign oral tumors, chronic periodontitis and healthy controls, using matrix-assisted laser desorption/ionization time-of-flight mass spectrometry (MALDI-TOF MS) and liquid chromatography tandem mass spectrometry. A principal component analysis plot showed distinct clusters among all groups. Four peptides were identified, including peptidyl-prolyl cis-trans isomerase FKBP4 isoform X2 (FKBP4), steroid hormone receptor ERR1 (ESRRA or ERRA), immunoglobulin superfamily member 10 (IGSF10) and ATP-binding cassette subfamily B member 5 (ABCB5). FKBP4, ESRRA and ABCB5 were found to be overexpressed in both LOM and LOSCC, whereas IGSF10 expression was markedly increased in LOSCC only. These four proteins also played a crucial role in numerous pathways of cancer metastasis and showed a strong relationship with chemotherapy drugs. In conclusion, this study showed rapid screening of canine oral tumors using serum and MALDI-TOF MS. In addition, potential serum peptidome-based biomarker candidates for LOM and LOSCC were identified.

## Introduction

Dogs can develop benign or malignant oral tumors, which represent nearly 6% of all canine cancers^1^. The most prevalent oral cancers in dogs include oral melanoma (OM), oral squamous cell carcinoma (OSCC), and fibrosarcoma^2^. For the benign oral tumors, acanthomatous ameloblastoma and peripheral odontogenic fibroma are commonly found^3^. According to the World Health Organization (WHO) clinical staging system for tumors of the oral cavity, stage I (a < 2 cm diameter tumor), stage II (a 2 to < 4 cm diameter tumor), stage III (a ≥ 4 cm tumor and/or lymph node metastasis) and stage IV (a tumor with distant metastasis) are characterized based on tumor size and metastasis^4^. Stages I and II are defined as an early-stage OM (EOM), whereas stages III and IV, which oral cancers are typically classified as, are defined as a late-stage OM (LOM)^5,6^. Normally, surgery and radiation can control the local invasiveness and improve the quality of a dog’s life. However, distant metastasis constitutes the major cause of oral cancer-related death^7,8^. Even though treatment options for OM are continually being developed, the survival time has not shown obvious improvement^9^.

Peptidomics has been used to identify several human cancer biomarkers, such as for colorectal cancer, early bladder cancer, prostate cancer, ovarian cancer, renal cell carcinoma and metastatic gastric cancer, using matrix-assisted laser desorption/ionization time-of-flight mass spectrometry (MALDI-TOF MS) with or without liquid chromatography tandem mass spectrometry (LC-MS/MS)^10–15^. In dogs, MALDI-TOF MS and in-gel digestion coupled with mass spectrometry (GeLC-MS/MS) has been applied to study peptidomic and proteomic profiles of canine oral tumor tissues and saliva^16,17^. However, a serum-based peptidomic biomarker of canine oral tumors has not yet been reported. Because of the highly metastatic behavior of canine oral cancers, novel circulating biomarkers can provide strong supporting evidence for diagnosis, prognosis, monitoring, and selecting treatment options. In addition, blood circulation supplies the organs and also tumors. Thus, blood sample is a widely useful source of circulating biomarkers. This study aimed to investigate the serum peptidome-based biomarkers of EOM, LOM, late-stage OSCC (LOSCC), benign oral tumors (BN), chronic periodontitis (PD) and healthy controls (CTRL), using MALDI-TOF MS and LC-MS/MS techniques.

## Results

### MALDI-TOF MS results

**Figure 1:**
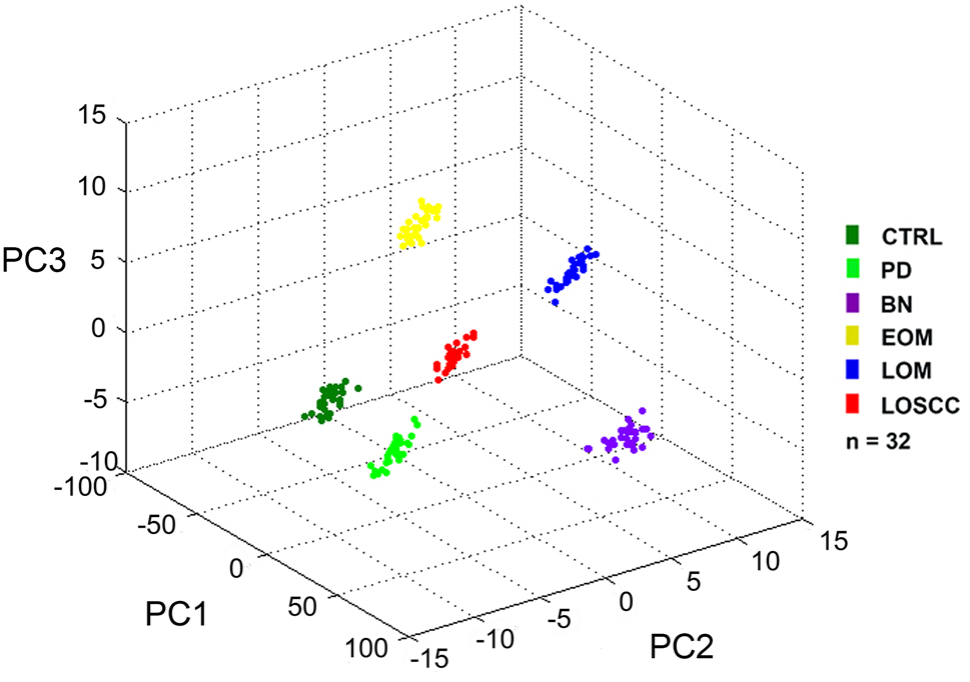
Three-dimensional principal component analysis scatterplot of normal (CTRL), periodontitis (PD), benign tumors (BN), early-stage oral melanoma (EOM), late-stage oral melanoma (LOM) and late-stage oral squamous cell carcinoma (LOSCC).

**Figure 2:**
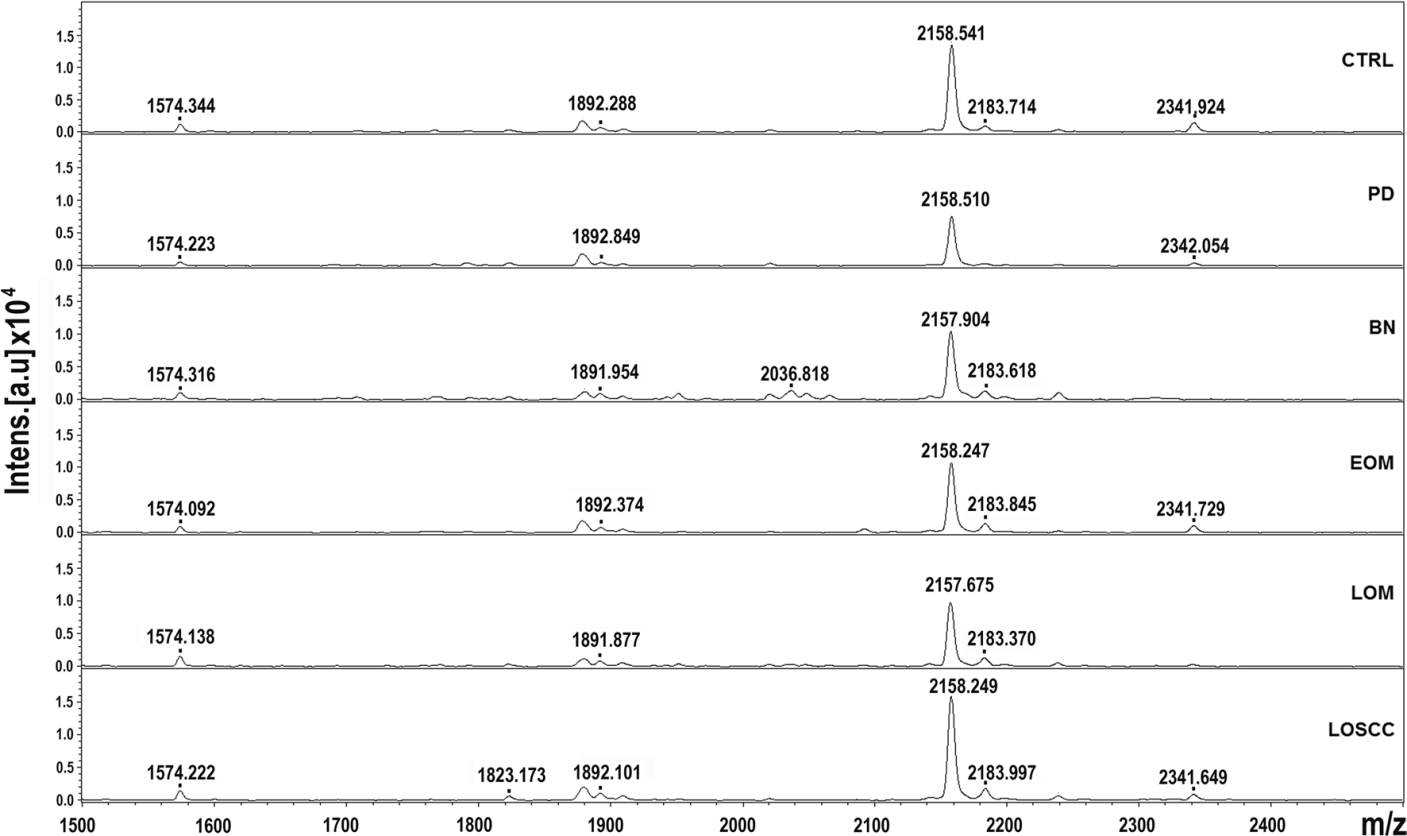
Peptide mass fingerprint of serum peptides from normal (CTRL), periodontitis (PD), benign tumors (BN), early-stage oral melanoma (EOM), late-stage OM (LOM) and late-stage oral squamous cell carcinoma (LOSCC) in the range 1500–2500 Da.

All 32 replicates in each pooled sample group demonstrated the homogeneity within the group. In the component analysis, ClinProTools revealed the different proteomic profiles from 1 to 20 kDa among 6 groups: CTRL, PD, BN, EOM, LOM and LOSCC. A 3-dimensional view of the principal component analysis (PCA) plot showed distinct clusters among these groups (Fig. 1). Therefore, there are differentially expressed protein and peptide profiles in each group. Peptide mass fingerprints (PMFs) from 1500 to 2500 Da were demonstrated (Fig. 2). In this mass range, significantly different peaks among the 6 groups could be detected.

**Figure 3:**
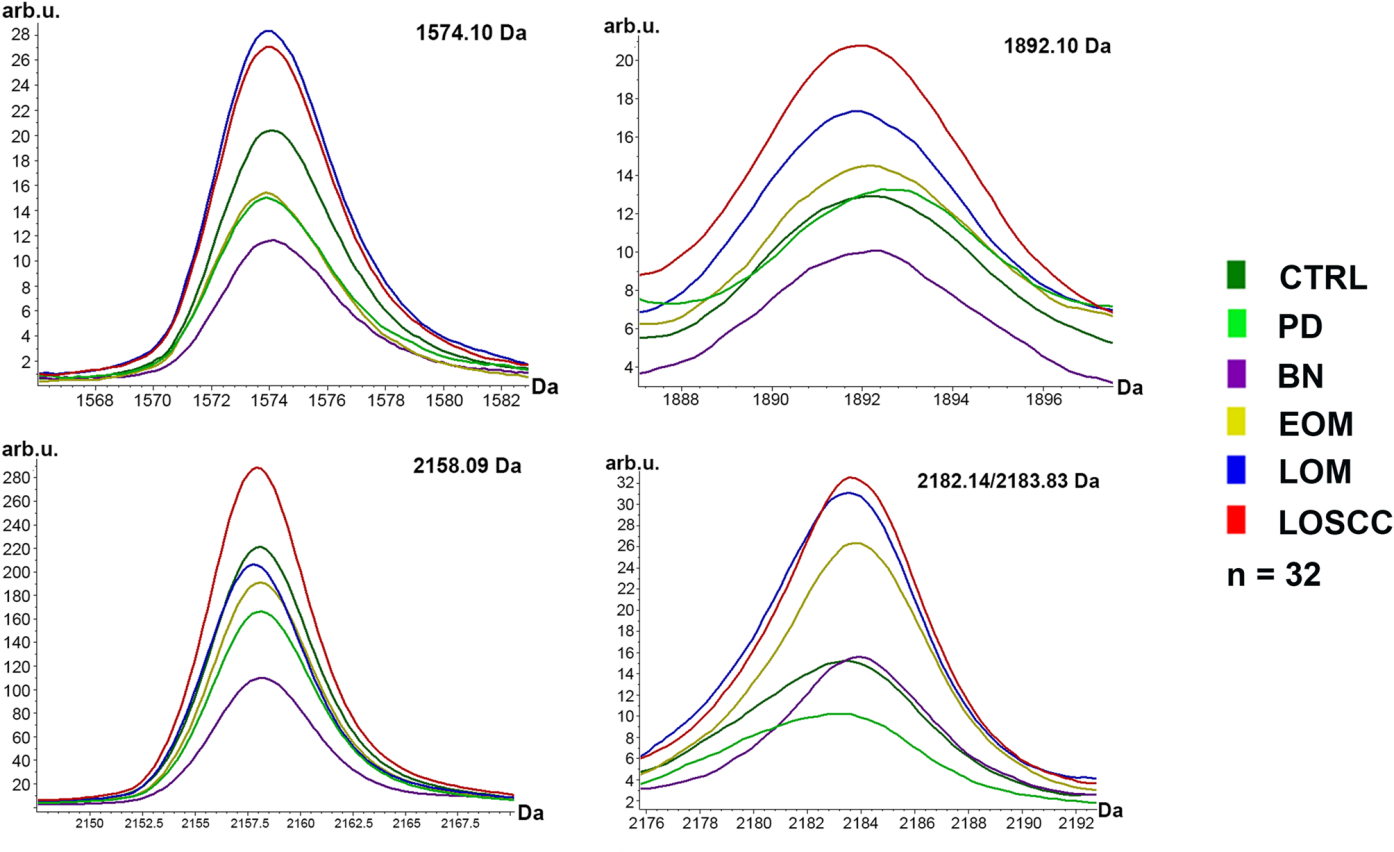
The significantly differentially overexpressed peptides in canine oral cancer groups. Normal (CTRL), periodontitis (PD), benign tumors (BN), early-stage oral melanoma (EOM), late-stage OM (LOM) and late-stage oral squamous cell carcinoma (LOSCC).

The MALDI-TOF MS results had an accurate outcome with the 95% confidence interval. The cross-validation and the recognition capability calculated by ANOVA and W/KW test in the EOM, LOM, LOSCC, BN, PD and CTRL groups was all 100%, indicating that the results were of high reliability. There were up to 19 different peaks identified among all analyzed groups, and four of them were significantly differentially overexpressed in canine oral cancer groups (EOM, LOM and LOSCC) with a mass ranging from m/z 1000 to 2500 Da, including m/z 1574.10, 1892.10, 2158.09 and 2182.14/2183.83 Da by ClinProTools software (Fig. 3). The peptide sequences of unique peaks were analyzed by LC-MS/MS.

## LC-MS/MS results

**Figure 4:**
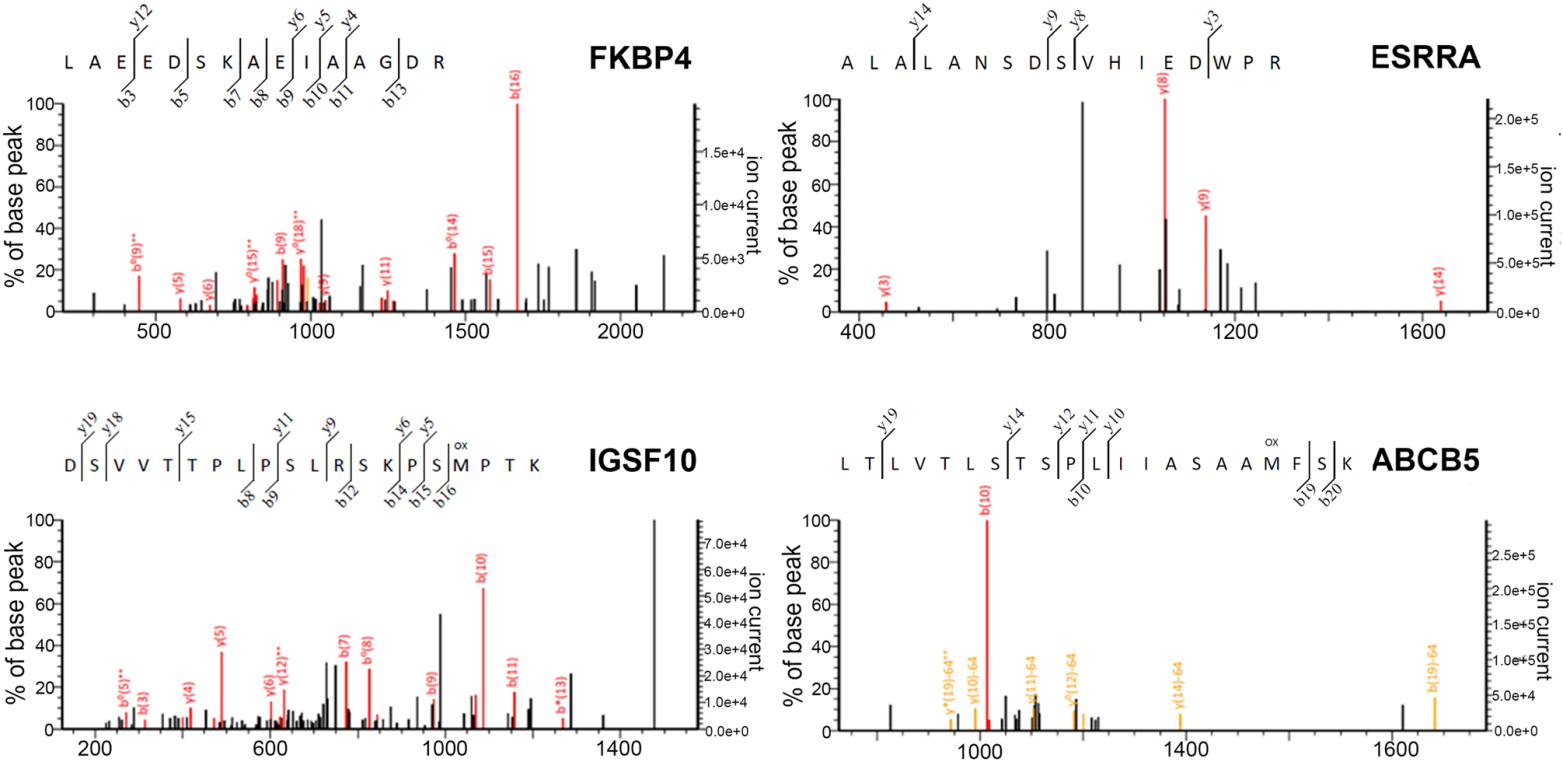
Analysis of four candidate peaks by LC-MS/MS. Peptidyl-prolyl cis-trans isomerase FKBP4 isoform X2 (FKBP4), steroid hormone receptor ERR1 (ESRRA), immunoglobulin superfamily member 10 (IGSF10) and ATP-binding cassette subfamily B member 5 (ABCB5).

The highest intensity of four peptide peaks (1574.10, 1892.10, 2158.09, and 2182.14/2183.83 Da) from cancerous groups appeared in the 18%, 24% and 26% ACN elution fractions (Table 1). The four peptides were sequenced and identified as peptidyl-prolyl cis-trans isomerase FKBP4 isoform X2 (FKBP4), steroid hormone receptor ERR1 (ESRRA, also known as ERRA), immunoglobulin superfamily member 10 (IGSF10) and ATP-binding cassette subfamily B member 5 (ABCB5) by MS/MS fragmentation, respectively. The results from Mascot software analysis are shown in Fig. 4. FKBP4, ESRRA and ABCB5 were found to be overexpressed in both LOM and LOSCC, whereas IGSF10 expression was markedly increased in LOSCC only. Candidate protein biomarkers were evaluated for biological processes and molecular function using UniProtKB/Swiss-Prot^18^ (Table 1).

**Table 1:** Candidate protein biomarkers were evaluated for biological processes and molecular function by UniProtKB/Swiss-Prot.

Unique marker (m/z)	Protein score	Sample groups	NCBI database	Peptide sequence	Protein name	Biological Process	Molecular function
1574.10	16	LOM & OSCC	XP_006862759.1	LAEEDSKAEIAAGDR	Peptidyl-prolyl cis–trans isomerase FKBP4 isoform X2 (FKBP4)	Androgen receptor signaling pathway, chaperone-mediated protein folding	ATP binding, FK506 binding, glucocorticoid receptor binding
1892.10	18	LOM & OSCC	XP_006176945.2	ALALANSDSVHIEDWPR	Steroid hormone receptor ERR1 (ESRRA)	DNA-binding transcription factor activity, steroid binding	Regulation of transcription, DNA-templated
2158.09	10	OSCC	XP_006994716.1	DSVVTTPLPSLRSKPSMPTK	Immunoglobulin superfamily member 10 (IGSF 10)	Regulation of neuron migration	Control of early migration of neurons expressing gonadotropin-releasing hormone
2182.14/2183.83	13	EOM, LOM & OSCC	XP_004418995.1	LTLVTLSTSPLIIASAAMFSK	ATP-binding cassette subfamily B member 5 (ABCB5)	Regulation of membrane potential, transmembrane transport	ATPase activity, ATPase-coupled transmembrane transporter activity

**Figure 5:**
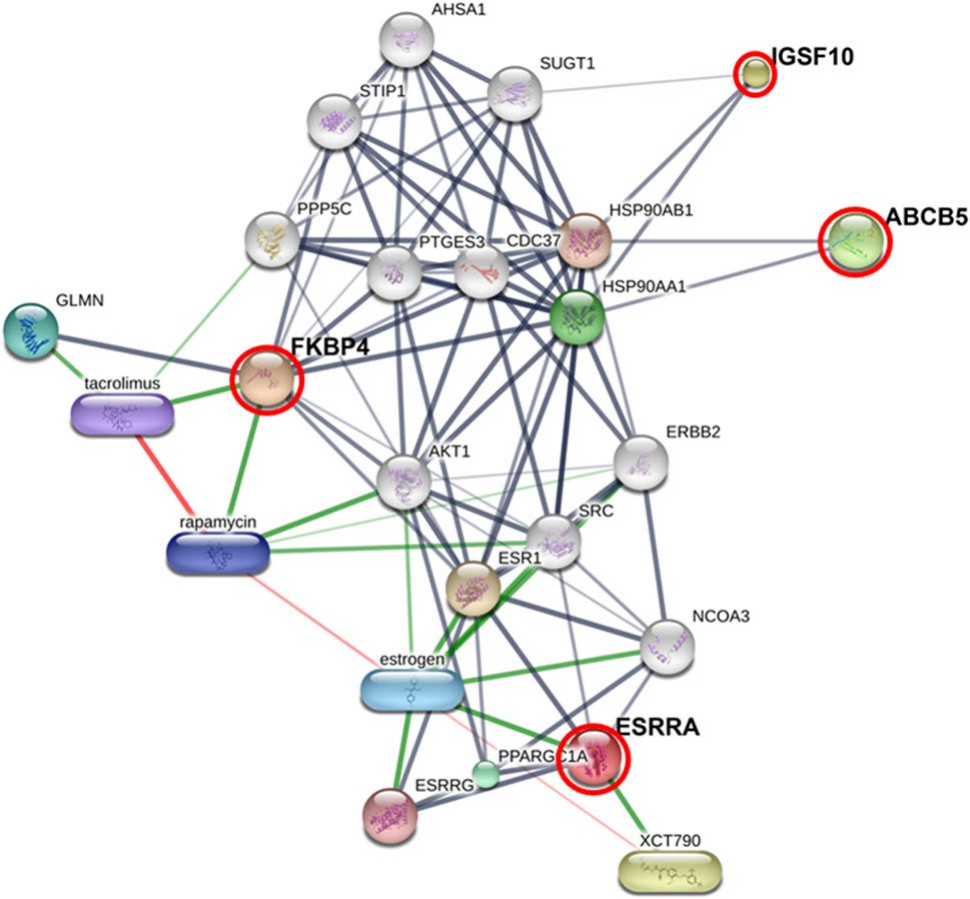
Involvement of peptidyl-prolyl cis-trans isomerase FKBP4 isoform X2 (FKBP4), steroid hormone receptor ERR1 (ESRRA), immunoglobulin superfamily member 10 (IGSF10) and ATP-binding cassette subfamily B member 5 (ABCB5) in networks of protein–chemical agent interactions based on KEGG pathways. Red circles: FKBP4, ESRRA, IGSF10 and ABCB5. Abbreviations: AHSA1, activator of heat shock 90 kDa protein ATPase homolog 1 (also known as AHA1); AKT1, v-akt murine thymoma viral oncogene homolog 1; CDC37, cell division cycle 37 homolog; ERBB2, v-erb-b2 erythroblastic leukemia viral oncogene homolog 2 (neuro/glioblastoma derived oncogene homolog); ESR1, estrogen receptor 1 (nuclear hormone receptor); ESRRG, estrogen-related receptor gamma; GLMN, glomulin (FKBP associated protein); HSP90AA1, heat shock protein 90 kDa alpha (cytosolic), class A member 1; HSP90AB1, heat shock protein 90 kDa alpha (cytosolic), class B member 1; NCOA3, nuclear receptor coactivator 3; PPARGC1A, peroxisome proliferator-activated receptor gamma, coactivator 1 alpha; PPP5C, protein phosphatase 5, catalytic subunit; PTGES3, prostaglandin E synthase 3 (cytosolic); SRC, v-src sarcoma (Schmidt-Ruppin A-2) viral oncogene homolog; STIP1, stress-induced-phosphoprotein 1; SUGT1, suppressor of G2 allele of SKP1 (also known as SGT1). Chemical agents: estrogen, rapamycin, tacrolimus and XCT790.

**Figure 6:**
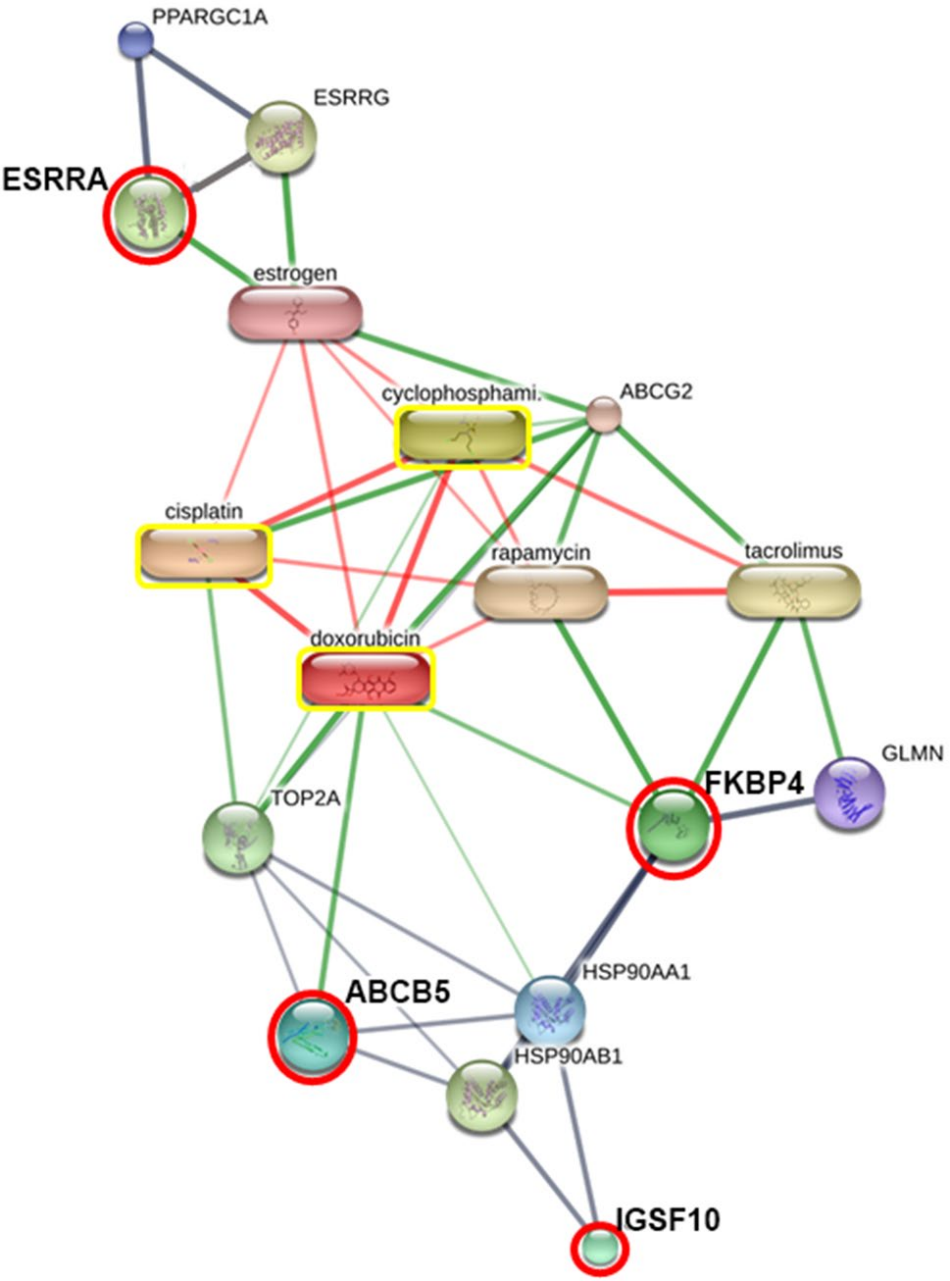
Involvement of peptidyl-prolyl cis–trans isomerase FKBP4 isoform X2 (FKBP4), steroid hormone receptor ERR1 (ESRRA), immunoglobulin superfamily member 10 (IGSF10) and ATP-binding cassette subfamily B member 5 (ABCB5) in networks of protein–chemotherapy drug interactions. Red circles: FKBP4, ESRRA, IGSF10 and ABCB5. Yellow boxes: doxorubicin, cisplatin and cyclophosphamide. Abbreviations: ABCG2, ATP-binding cassette, subfamily G (WHITE), member 2; ESRRG, estrogen-related receptor gamma; GLMN, glomulin (FKBP associated protein); HSP90AA1, heat shock protein 90 kDa alpha (cytosolic), class A member 1; HSP90AB1, heat shock protein 90 kDa alpha (cytosolic), class B member 1; PPARGC1A, peroxisome proliferator-activated receptor gamma, coactivator 1 alpha; TOP2A, topoisomerase (DNA) II alpha 170 kDa. Chemical agents: estrogen, rapamycin and tacrolimus.

The protein interactions network of FKBP4, ESRRA, IGSF10 and ABCB5 showed a strong relationship with chemotherapy drugs, including doxorubicin, cisplatin, and cyclophosphamide and also with their predicted functional partners, including heat shock protein 90 alpha family class A member 1 (HSP90AA1), peroxisome proliferator-activated receptor, gamma, coactivator 1 alpha (PPARGC1A), glomulin (GLMN), FKBP-associated protein, estrogen, rapamycin, tacrolimus, estrogen-related receptor gamma (ESRRG), heat shock protein 90 alpha family class B member 1 (HSP90AB1), estrogen receptor 1 (ESR1) and XCT790, using STITCH 5.0 (Figs. 5 and 6). These proteins have been reported to play a crucial role in numerous pathways of cancer metastasis as shown in Kyoto Encyclopedia of Genes and Genomes (KEGG) pathway maps, namely nucleotide-binding oligomerization domain (NOD)-like receptor signaling pathway, ErbB signaling pathway, PI3K-Akt signaling pathway, vascular endothelial growth factor (VEGF) signaling pathway, and adherens junctions^19^.

## Discussion

In the present study, PMFs and peptidomic clusters were obtained from the serum of canine oral tumors. In addition, FKBP4, ESRRA and ABCB5 were found to be overexpressed in both LOM and LOSCC, whereas IGSF10 expression was markedly increased in LOSCC only.

FKBPs are members of the immunophilin families. A high level of FKBP4 was demonstrated in several human cancers, including breast cancer, ovarian cancer and osteosarcoma, using proteomic techniques^20–23^. In addition, marked expression of FKBPs was noted in other human cancers, such as cutaneous melanoma, glioma, prostate cancer, lymphoma and hepatocellular carcinoma^24–28^. Recently, FKBP4 has been reported to be a novel PI3K/PDK1/mTORC2 interacting protein and promoted Akt phosphorylation through PI3K/PDK1 and mTORC2. Since the PI3K/PDK1/Akt signaling pathway activates and regulates cell proliferation, cell cycle and survival, FKBP4 is directly related to cell growth promotion and proliferation^21^ (Fig. 5). With regard to ESRRA, it is an orphan nuclear receptor that was shown to be activated by peroxisome proliferator activated receptor gamma coactivator-1 alpha and beta (PGC-1a and PGC-1b)^29^. Several enzymes in metabolic pathways of proliferating cancer cells control the transcription of ESRRA, depending on the energy requirement^30,31^. In previous studies, overexpression of ESRRA was exhibited to promote cancer cell growth and proliferation^30,32,33^. Moreover, the epidermal growth factor/Erb-B2 receptor tyrosine kinase 2 (EGF/ERBB2) signaling pathway in breast cancer cells modulated the transcription activity of ESRRA, which was associated with poor prognosis in breast and ovarian cancers^32,34,35^. Implanted xenografts with knockdown of ESRRA expression in MDA-MB-231 cells can reduce the tumor growth rate of breast cancers^36^. In the present study, we found prominent expression of FKBP4 and ESRRA in LOM and LOSCC, possibly being novel prognostic biomarkers and therapeutic target candidates. Hence, their role in cancer cell growth and proliferation in LOM and LOSCC should be further investigated.

For ABCB5 protein, ABCB5 is an ABC transporter and P-glycoprotein family member^37^. This protein had been reported to be expressed in human cutaneous melanoma and be associated with chemoresistance^38,39^. Moreover, it was upregulated in circulating melanoma tumor cells and correlated with pulmonary metastasis formation in human-to-mouse subcutaneous tumor xenotransplantation models^40^. In our present study, increased expression of ABCB5 in LOM and OSCC might represent a biomarker associated with stages of metastasis, as per a previous report^40^. For IGSF10, IGSF is classified based on molecules combined with immunoglobulins. IGSF10, a member of the IGSF, is reported to control the early migration of gonadotropin-releasing hormone expressing neurons and maintenance of the osteochondroprogenitor cell pool^41^. Inhibition of IGSF10 expression by IGSF10-small interfering RNA was demonstrated to inhibit cancer cell proliferation, enhance adhesion between cells and matrix, and increase the survival rate of patients with lung cancer by activating the integrin-β1/FAK pathway^42^. We demonstrated very high and unique expression of IGSF10 in LOSCC, which revealed a potential biomarker candidate of LOSCC. In addition, its role in carcinogenesis and cancer progression should be further investigated.

Because of the high rate of metastasis and aggressiveness of melanoma in dogs, systemic medication such as chemotherapy should be used as a treatment option^2^. In the present study, using Stitch EMBL, the interaction network analysis revealed that a coordination of candidate proteins (FKBP4, ESRRA, IGSF10 and ABCB5) was involved in KEGG pathways of many cancers (Fig. 5). In addition, the four proteins appeared to link with chemotherapy drugs (carboplatin, doxorubicin and cyclophosphamide), which could probably lead to the development of serum biomarker-based liquid biopsy for efficient diagnosis, prognosis, recurrence and metastasis predictions (Fig. 6). The networks of the candidate proteins and the chemotherapy drugs showed a strong relationship with DNA topoisomerase II alpha (TOP2A), a biomarker of oral cancers (Fig. 6)^43,44^. The association of target proteins with TOP2A, chemotherapy drugs, particularly carboplatin, doxorubicin and metronomic chemotherapy based on cyclophosphamide, and/or immunosuppression agents such as rapamycin and tacrolimus should be further investigated. This might be of great benefit for veterinarians and researchers to understand the pathogenesis and biological process of canine oral cancer.

MALDI-TOF MS was used to discover several specific serum peptide patterns of human cancers as well as tissues and whole saliva of canine oral tumors^16,17,45,46^. Since cancer cells usually invade blood vessels to metastasize to other distant organs, serum samples are of interest for exploring novel metastatic biomarkers^47–49^. To the best of our knowledge, this is the first publication of serum proteomics in canine oral tumors. Using MALDI-TOF MS, we found distinct PMF patterns among CTRL, PD, BN, EOM, LOM and LOSCC groups. Therefore, this instrument could be considered as a high sensitivity diagnostic tool for rapid screening for canine oral tumors. Using LC-MS/MS, four peptide regions of FKBP4, ESRRA, ABCB5 and IGSF10 were identified in LOSCC and/or LOM. For dogs with recurrence or metastatic stages following surgery, the diagnosis and treatment are usually too late because the current metastasis stage diagnosis relies on cytology of regional lymph nodes and imaging analysis (X-rays, ultrasound, and computerized tomography scan). Our studies could probably help researchers to better understand, and encourage future studies of, disease recurrence and spreading following treatment.

## Conclusion

Our data suggested that MALDI-TOF MS used with ClinProTools software has potential to be used for rapid screening in serum of canine oral tumors. The peptide fragment of serum proteins was identified using MALDI-TOF MS and LC-MS/MS, combined with the C18 ZipTip technique. FKBP4, ESRRA, IGSF10 and ABCB5 might be potential biomarkers for diagnosis, prognosis and metastasis prediction. The interaction network showed the proteins and chemotherapy drugs that were involved in the biological process and pathogenesis of canine oral cancer. Further studies should be performed in a larger population to evaluate these potential candidate biomarkers.

## Materials and methods

### Ethics statement

All methods were carried out in accordance with guidelines and regulations, and the study was performed in compliance with the ARRIVE guidelines. All experimental protocols were approved by Mahidol University-Animal Care and Use Committee (MUVS-ACUC), Faculty of Veterinary Science, Mahidol University, Thailand (Protocol No. MUVS-2021-04-14). All procedures were carried out in accordance with the relevant guidelines and regulations. Samples were obtained with the informed consent of owners.

### Animals and sampling

All samples were collected from the Prasu-Arthorn Animal Hospital, Faculty of Veterinary Science, Mahidol University and the Small Animal Teaching Hospital, Faculty of Veterinary Science, Chulalongkorn University, Thailand. Dogs with oral tumors received oral and physical examination; moreover, the metastasis was determined by cytological examination, skull to abdomen radiography and abdominal ultrasound. The stages of oral cancer were determined according to WHO classification and diagnosed following the results of veterinary pathology. All patients with any other tumors and those with prescription chemotherapy were excluded. The study protocol was approved by the Mahidol University-Animal Care and Use Committee (MUVS-ACUC), Thailand (Protocol No. MUVS-2021-04-14).

The total of 67 serum samples were collected from the following: 5 EOM, 28 LOM, 10 LOSCC, 12 BN (age range: 7–14 years) and 5 dogs with PD (age range: 7–13 years); and samples representing normal oral health were gathered from 7 healthy dogs (CTRL) (range: 7–8 years) (Table 2). The PD group was diagnosed by oral examination with appearance of gingivitis, dental tartar and loss of periodontal attachment. Whole blood (~ 3–4 mL) was collected from the cephalic or the saphenous vein. The collected blood was centrifuged at 3000*g* at 4 °C for 15 min. Serum samples (~ 500 µL) were mixed with protease inhibitor cocktails (Thermo Fisher Scientific) and stored at − 20 °C until use. Using bovine serum albumin as a standard, total protein concentrations from serum samples were evaluated by Lowry’s assay.

**Table 2:** Patients’ history.

Group^a^	Clinical examination/histological finding	Breed	Ages (yrs) Mean ± SD	Sex^b^
CTRL	Normal gingiva	7 Beagles	7.85 ± 1.46	4 M and 3 Fs
PD	Periodontitis	2 Poodles and 3 Mixed	8.67 ± 1.21	3 Mc and 2 Fs
BN	Peripheral odontogenic fibroma	2 Poodles, 3 Golden Retrievers, 2 Siberian huskies, 1 Mixed and 1 Shi-tsu	8.75 ± 3.14	4 M, 2 Mc, 1 F and 2 Fs
	Acanthomatous ameloblastoma	1 Shi-tsu, 1 Labrador retriever and 1 Golden Retriever	10.67 ± 0.58	2 F and 1 Fs
EOM	Melanotic melanoma	1 Poodle, 1 Mixed and 1 Chihuahua	10.33 ± 0.58	3 M
	Amelanotic melanoma	1 Poodle and 1 Mixed	13 ± 1.41	2 M
LOM	Melanotic melanoma	3 Poodles, 5 Golden Retrievers, 4 Mixed, 1 Pug, 2 Shi-tsus, 1 Labrador retriever, 1 Beagle, 1 Cocker spaniel, 1 Schnauzer and 1 Pomeranian	12.61 ± 2.15	14 M, 2 F and 4 Fs
	Amelanotic melanoma	1 Poodle, 1 Cocker spaniel, 5 Mixed and 1 Dachshund	10.67 ± 1.75	5 M, 2 Mc and 1 F
LOSCC	OSCC well differentiated	1 Poodle, 1 Cocker spaniel, 2 Mixed and 1 Pug	12 ± 1.26	2 M, 1 F and 3 Fs
	OSCC poorly differentiated	1 Poodle, 1 Mixed, 1 Shi-tsu and 1 Bangkeaw	11.75 ± 2.5	2 M, 1 Mc and 1 F

^a^*CTRL* control, *PD* periodontitis, *BN* benign tumors, *EOM *early-stage oral melanoma, *LOM* late-stage oral melanoma, *LOSCC* late-stage oral squamous cell carcinoma.

^b^*M* male, *Mc* male castration, *F* female, *Fs* female spray.

### Peptide analysis by MALDI-TOF MS

Sixty-seven samples were prepared with 0.1% trifluoroacetic acid (TFA) for analysis to a final concentration of 0.1 µg/µL. One microliter of a peptide, mixed with a matrix solution (5 mg/mL α-cyano-4-hydroxycinnamic acid (CHCA) in 100% acetonitrile (ACN) containing 5% TFA), was directly spotted onto the MTP384 target plate (Bruker Daltonics, Billerica, Massachusetts, USA) in eight replicates to prevent sample preparation bias. Target samples were tested using an Ultraflex III TOF/TOF system (Bruker Daltonics), using a linear positive mode with a mass range 1000–5000 Da. Each spectrum was obtained from 500 laser shots, with a 50 Hz laser. A ProteoMass peptide and protein MALDI MS calibration kit (Sigma Aldrich, St. Louis, Missouri, USA) was used for external calibration. MS spectra and peptide patterns were analyzed by flexAnalysis version 3.2 and ClinProTool version 3.0 software (Bruker Daltonics), respectively. The results were processed by a standard workflow that comprised fingerprint spectra, pseudo-gel view, dendrogram and PCA. Four from eight replicates of each sample were selected and pooled, together with other samples in each group. Next, 32 replicates of pooled samples were applied twice to MALDI-TOF MS to prevent sample preparation bias in each group. After the analysis by flexAnalysis and ClinProTool software was performed, the data were processed for peak selection and peak calculation. The recognition capability and cross-validation values of more than 90% exhibited the reliability of the peak selection^50^. ClinProTools v. 3.0 was used to analyze intensity values. Results with *P* < 0.05 were considered significant. Subsequently, the selected peptides from pooled samples in each group were purified, using C18 ZipTip (Merck Millipore, Burlington, MA, USA). Each sample was eluted in a 2% series of ACN in the range 0–100%. The second MALDI-TOF MS was performed for eluted fractions as mentioned above. The intensity of MS spectra was calculated by flexAnalysis (Bruker Daltonics). The fractions of selected peptides with the highest intensity were collected for further LC-MS/MS analysis.

### Peptide identification by LC-MS/MS

The selected peptide fractions were sequenced by reversed-phase high performance liquid chromatography and a PTM Discovery System (Bruker Daltonics) coupled to an UltiMate 3000 LC System (Thermo Fisher Scientific, Waltham, MA). A quadrapole ion-trap MS instrument (Bruker Daltonics) was used to analyze the serum peptides. Before analysis, a PepSwift monolithic nanocolumn (100 μm diameter × 50 mm length) was used to separate serum peptides based on differential polarity and molecular weight. The column was connected to electrospray ionization in the positive ion mode. Mobile phase A and B were prepared from 0.1% formic acid dilution and from 50% ACN in water containing 0.1% formic acid, respectively. A flow rate of 1000 nL/min was set to separate peptides with a 4–70% linear gradient of mobile phase B. Next, the peptides were regenerated and equilibrated using 90% and 4% eluent B, respectively, for 20 min per run. Peptide fragment mass spectra were selected from the MS scan at 200–2800 m/z. AutoMS mode was used to analyze peptide spectra with a scan range of 400–1500 m/z, 3 averages, and up to 5 precursor ions. The results of LC-MS/MS were converted into an mzXML file by CompassXport software (Bruker Daltonics). Protein was quantified using DeCyder MS Differential Analysis software (Amersham Biosciences, Little Chalfont, UK)^51,52^. The PepDetect module was used in MS mode for automated peptide detection, charge state assignments, and peptide ion signal intensities. The peptides were identified from MS/MS peptide mass values using Mascot software (Matrix Science, London, UK)^53^. The information about detailed analysis of the protein sequences and biological process was used in the annotation of UniProtKB/Swiss-Prot entries^18^. Stitch 5.0 (http://stitch.embl.de) was performed automatically to elucidate interactions of target proteins-other proteins, chemotherapeutic drugs and/or immunosuppressive drugs^54^.

### Statistical analyses

To analyze intensity values of PMF spectra, two statistical tests, t-test/ANOVA (TTA) and Wilcoxon/Kruskal–Wallis (W/KW), incorporated into ClinProTools software version 3.0, were used. For LC-MS/MS analysis, ANOVA statistical analysis incorporated into the DeCyder MS was used to identify significantly varying peptides among different sample groups. Significance was accepted at the *P* < 0.05 level.

### Ethics approval and consent to participate

The study was approved by the Mahidol University-Animal Care and Use Committee (MUVS-ACUC), Thailand (Protocol No. MUVS-2021-04-14).

## Data Availability

The raw MS/MS spectra data are available in ProteomeXchange: JPST001868 and PXD037051. (https://repository.jpostdb.org/preview/803629563355f75c5eb3) Access key: 8198.

## References

[1] North SM, Banks TA (2009). Tumours of head and neck. Small Anim. Oncol..

[2] Liptak JM, Withrow SJ, Withrow M (2013). Cancer of the gastrointestinal tract. Withrow and MacEwen’s Small Animal Clinical Oncology.

[3] Cray M, Selmic LE, Ruple A (2020). Demographics of dogs and cats with oral tumors presenting to teaching hospitals: 1996–2017. J. Vet. Sci..

[4] Owen L (1980). TNM Classification of Tumours in Domestic Animals.

[5] Smith SH, Goldschmidt MH, McManus PM (2002). A comparative review of melanocytic neoplasms. Vet. Pathol..

[6] Pisamai S, Rungsipipat A, Kalpravidh C, Suriyaphol G (2017). Gene expression profiles of cell adhesion molecules, matrix metalloproteinases and their tissue inhibitors in canine oral tumors. Res. Vet. Sci..

[7] Bergman PJ (2007). Canine oral melanoma. Clin. Tech. Small Anim. Pract..

[8] Chen TC (2013). The impact of perineural invasion and/or lymphovascular invasion on the survival of early-stage oral squamous cell carcinoma patients. Ann. Surg. Oncol..

[9] Pazzi P, Steenkamp G, Rixon AJ (2022). Treatment of canine oral melanomas: a critical review of the literature. Vet. Sci..

[10] Wang H (2017). Serum peptidome profiling for the diagnosis of colorectal cancer: Discovery and validation in two independent cohorts. Oncotarget.

[11] Ding D, Chen M, Xiao X, Cao P, Li S (2020). Novel serum peptide model revealed by MALDI-TOF-MS and its diagnostic value in early bladder cancer. Int. J. Biol. Markers.

[12] Padoan A (2018). MALDI-TOF peptidomic analysis of serum and post-prostatic massage urine specimens to identify prostate cancer biomarkers. Clin. Proteomics.

[13] Swiatly A (2017). MALDI-TOF-MS analysis in discovery and identification of serum proteomic patterns of ovarian cancer. BMC Cancer.

[14] Gianazza E (2012). Alterations of the serum peptidome in renal cell carcinoma discriminating benign and malignant kidney tumors. J. Proteom..

[15] Abramowicz A (2015). Identification of serum proteome signatures of locally advanced and metastatic gastric cancer: A pilot study. J. Transl. Med..

[16] Pisamai S, Roytrakul S, Phaonakrop N, Jaresitthikunchai J, Suriyaphol G (2018). Proteomic analysis of canine oral tumor tissues using MALDI-TOF mass spectrometry and in-gel digestion coupled with mass spectrometry (GeLC MS/MS) approaches. PLoS ONE.

[17] Ploypetch S (2019). Salivary proteomics of canine oral tumors using MALDI-TOF mass spectrometry and LC-tandem mass spectrometry. PLoS ONE.

[18] UniProt Consortium, T (2018). UniProt: The universal protein knowledgebase. Nucl. Acids Res..

[19] Kanehisa M, Goto S (2000). KEGG: Kyoto encyclopedia of genes and genomes. Nucl. Acids Res..

[20] Lawrenson K (2015). Identification of novel candidate biomarkers of epithelial ovarian cancer by profiling the secretomes of three-dimensional genetic models of ovarian carcinogenesis. Int. J. Cancer.

[21] Mangé A (2019). FKBP4 connects mTORC2 and PI3K to activate the PDK1/Akt-dependent cell proliferation signaling in breast cancer. Theranostics.

[22] Niforou KM (2008). The proteome profile of the human osteosarcoma U2OS cell line. Cancer Genom. Proteom..

[23] Yang WS (2012). Proteomic approach reveals FKBP4 and S100A9 as potential prediction markers of therapeutic response to neoadjuvant chemotherapy in patients with breast cancer. J. Proteome Res..

[24] Romano MF (2004). Rapamycin inhibits doxorubicin-induced NF-κB/Rel nuclear activity and enhances the apoptosis of melanoma cells. Eur. J. Cancer.

[25] Solassol J, Mange A, Maudelonde T (2011). FKBP family proteins as promising new biomarkers for cancer. Curr. Opin. Pharmacol..

[26] Febbo PG (2005). Androgen mediated regulation and functional implications of FKBP51 expression in prostate cancer. J. Urol..

[27] Baughman G, Wiederrecht GJ, Campbell NF, Martin MM, Bourgeois S (1995). FKBP51, a novel T-cell-specific immunophilin capable of calcineurin inhibition. Mol. Cell. Biol..

[28] Liu H (2015). PTP1B promotes cell proliferation and metastasis through activating src and ERK1/2 in non-small cell lung cancer. Cancer Lett..

[29] Deblois G, St-Pierre J, Giguère V (2013). The PGC-1/ERR signaling axis in cancer. Oncogene.

[30] Deblois G, Giguère V (2013). Oestrogen-related receptors in breast cancer: control of cellular metabolism and beyond. Nat. Rev. Cancer.

[31] Stein RA (2008). Estrogen-related receptor α is critical for the growth of estrogen receptor–negative breast cancer. Cancer Res..

[32] Fujimoto J (2007). Clinical implication of estrogen-related receptor (ERR) expression in ovarian cancers. J. Steroid Biochem. Mol. Biol..

[33] Teng J, Wang Z-Y, Jarrard DF, Bjorling DE (2008). Roles of estrogen receptor and in modulating urothelial cell proliferation. Endocr. Relat. Cancer.

[34] Ariazi EA, Kraus RJ, Farrell ML, Jordan VC, Mertz JE (2007). Estrogen-related receptor α1 transcriptional activities are regulated in part via the ErbB2/HER2 signaling pathway. Mol. Cancer Res..

[35] Suzuki T (2004). Estrogen-related receptor α in human breast carcinoma as a potent prognostic factor. Cancer Res..

[36] Deblois G, Giguère V (2011). Functional and physiological genomics of estrogen-related receptors (ERRs) in health and disease. Biochim. Biophys. Acta.

[37] Chen KG (2005). Principal expression of two mRNA isoforms (ABCB 5α and ABCB 5β) of the ATP-binding cassette transporter gene ABCB 5 in melanoma cells and melanocytes. Pigment Cell Res..

[38] Chen KG, Valencia JC, Gillet J-P, Hearing VJ, Gottesman MM (2009). Involvement of ABC transporters in melanogenesis and the development of multidrug resistance of melanoma. Pigment Cell Melanoma Res..

[39] Frank NY (2005). ABCB5-mediated doxorubicin transport and chemoresistance in human malignant melanoma. Cancer Res..

[40] Ma J (2010). Isolation of tumorigenic circulating melanoma cells. Biochem. Biophys. Res. Commun..

[41] Howard SR (2016). IGSF10 mutations dysregulate gonadotropin-releasing hormone neuronal migration resulting in delayed puberty. EMBO Mol. Med..

[42] Ling B (2020). Identification of prognostic markers of lung cancer through bioinformatics analysis and in vitro experiments. Int. J. Oncol..

[43] Di Leo A (2011). HER2 and TOP2A as predictive markers for anthracycline-containing chemotherapy regimens as adjuvant treatment of breast cancer: A meta-analysis of individual patient data. Lancet Oncol..

[44] Moelans CB, de Weger RA, van Blokland MT, van der Wall E, van Diest PJ (2010). Simultaneous detection of TOP2A and HER2 gene amplification by multiplex ligation-dependent probe amplification in breast cancer. Mod. Pathol..

[45] Han K (2008). Identification of lung cancer patients by serum protein profiling using surface-enhanced laser desorption/ionization time-of-flight mass spectrometry. Am. J. Clin. Oncol..

[46] Pietrowska M (2009). Mass spectrometry-based serum proteome pattern analysis in molecular diagnostics of early-stage breast cancer. J. Transl. Med..

[47] Zhou J (2017). Soluble PD-L1 as a biomarker in malignant melanoma treated with checkpoint blockade. Cancer Immunol. Res..

[48] Iivanainen S, Ahvonen J, Knuuttila A, Tiainen S, Koivunen JP (2019). Elevated CRP levels indicate poor progression-free and overall survival on cancer patients treated with PD-1 inhibitors. ESMO Open.

[49] Hardy-Werbin M (2019). Serum cytokine levels as predictive biomarkers of benefit from ipilimumab in small cell lung cancer. Oncoimmunology.

[50] Niyompanich S, Srisanga K, Jaresitthikunchai J, Roytrakul S, Tungpradabkul S (2015). Utilization of whole-cell MALDI-TOF mass spectrometry to differentiate *Burkholderia pseudomallei* wild-type and constructed mutants. PLoS ONE.

[51] Johansson C (2006). Differential expression analysis of *Escherichia coli* proteins using a novel software for relative quantitation of LC-MS/MS data. Proteomics.

[52] Thorsell A, Portelius E, Blennow K, Westman-Brinkmalm A (2007). Evaluation of sample fractionation using micro-scale liquid-phase isoelectric focusing on mass spectrometric identification and quantitation of proteins in a SILAC experiment. Rapid Commun. Mass Spectrom..

[53] Perkins DN, Pappin DJC, Creasy DM, Cottrell JS (1999). Probability-based protein identification by searching sequence databases using mass spectrometry data. Electrophoresis.

[54] Szklarczyk D (2016). STITCH 5: Augmenting protein–chemical interaction networks with tissue and affinity data. Nucl. Acids Res..

